# Auxin-Producing Bacteria Used as Microbial Biostimulants Improve the Growth of Tomato (*Solanum lycopersicum* L.) Seedlings in Hydroponic Systems

**DOI:** 10.3390/biotech13030032

**Published:** 2024-08-21

**Authors:** Livia Pappalettere, Susanna Bartolini, Annita Toffanin

**Affiliations:** 1 Institute of Crop Science, Sant’Anna School of Advanced Studies, Piazza Martiri della Libertà 33, 56127 Pisa, Italy; susanna.bartolini@santannapisa.it; 2 Department of Agriculture Food and Environment (DAFE), University of Pisa, Via del Borghetto 80, 56124 Pisa, Italy; annita.toffanin@unipi.it; 3 CIRSEC (Centro Interdipartimentale per lo Studio degli Effetti del Cambiamento Climatico dell’Università di Pisa), Centre for Climate Change Impact, University of Pisa, Via del Borghetto 80, 56124 Pisa, Italy

**Keywords:** endophytic bacteria, *Azospirillum* spp., *Bacillus* spp., hydroponic, IAA, rhizobacteria, in situ staining, PGPB, tomato, Canestrino di Lucca, inoculation method

## Abstract

Seven auxin-producing endophytic bacterial strains (*Azospirillum* spp., *Methylobacterium symbioticum*, *Bacillus* spp.), and two different combinations of these strains were used to verify their influence on tomato during germination and development in hydroponic conditions where, as a novelty for Canestrino di Lucca cultivar, endophytic bacteria were inoculated. To emphasize the presence of bacterial auxins in roots and stems of seedlings, both in situ staining qualitative assessment and quantitative analysis were carried out. Moreover, hypogeal and epigeal growth of the plantlets were measured, and correlation analyses were conducted to examine the relationship between the amount of indolacetic acid (IAA) produced by the bacterial strains and root and stem parameters. Plantlets treated with microbial inoculants showed a significant increase in the survival rate compared to the control treatment. The best results as IAA producers were from *Azospirillum baldaniorum* Sp245 and *A. brasilense* Cd, which also induced significant root growth. On the other hand, *Bacillus amyloliquefaciens* and *B. licheniformis* induced the best rates in stem growth. These findings highlight the potential for using endophytic bacterial strains in a hydroponic co-cultivation system that enables inoculating plantlets, at an early stage of growth (5 days old).

## 1. Introduction

Global warming, a decline in farming regions, water scarcity, and population expansion are major issues affecting food production worldwide [1]. Plant growth suffers by soil contamination for pathogens and toxicants, inadequate humus content, acidic or alkaline soil pH, insufficient drainage, land deterioration and adverse weather conditions (i.e., drought and floods). Weather conditions affect crop yield, and droughts and floods can cause a large portion of the harvest to be lost [2,3,4]. In alternative to conventional soil-based agriculture, the hydroponic system, using mineral nutrient solutions as growth substrate for plants, enables avoiding the above-mentioned drawbacks. This technique, occasionally supported with natural or synthetic materials, is more economical and ecologically friendly, producing healthier plants in comparison with traditional cultivations [5].

The majority of hydroponic systems (HS) have been utilized for commercial vegetable and cut flower production and they are also frequently employed for gardening, education, and research [6]. Among vegetables, tomatoes make considerable use of HS [7]. Compared to open-field cultivation, growing tomatoes in HS offers several advantages: increased crop yield and plant density per square meter, improved water use efficiency, and a reduced carbon footprint [8].

Growers can choose from a wide variety of HS options, but more innovations are required to improve crop yields and profitability. The cost of fertilizers has risen dramatically. Since 2021, prices rose by 78.6% over the previous year [9]. Furthermore, nitrogen fertilizers, which are widely used in HS (especially for tomatoes), result in higher N_2_O emissions compared to traditional open-field cultivation [10,11,12]. Thus, finding solutions that will enhance HS production and decrease its environmental and cost impact is a current topic. Developing microbial inoculants containing plant growth-promoting bacteria (PGPB) that are able to promote plant growth is considered an interesting solution [13].

In soil-based systems, PGPB provide substantial advantages to plants. This involves facilitating the plant’s uptake of nutrients, boosting growth, mitigating stress, and preventing infections. PGPB make it easier for plants to absorb vital nutrients from soil or atmosphere (such as iron, phosphorus, potassium, and fixed nitrogen). In addition, certain PGPB synthesize phytohormones such as auxins, cytokinins, and gibberellins, which have a direct impact on plant growth [14].

The use of plant growth-promoting bacteria (PGPB) in hydroponic systems is considered a sustainable and efficient alternative to chemical fertilizers [15]. Unlike chemical fertilizers, which can lead to nutrient runoff and environmental pollution, PGPB can establish a symbiotic relationship with plants, optimizing nutrient uptake and improving overall plant health, providing protection against pathogens and abiotic stressors. In addition, the utilization of PGPB within a controlled growth system (such as HS) can enhance PGPB effectiveness and prolong microbial activity, since it lacks the intense competition often found in the rhizosphere [13]. Although research on this topic is still limited, the existing studies yield encouraging results. Gul [16] reported that in a hydroponic tomato system, plants treated with PGPB produced a higher yield than the control treatment, as PGPB supported plant growth and biological control efficacy. Furthermore, Aini [17] have verified that tomato plants grown in HS treated with biological agents produced fruits that weighed more than those that were not.

A HS containing N-fixing endophytic bacteria that can supply nitrogen through plant tissues, can also decrease the need for inorganic nutrients [18]. Examples of these PGPB include *Azospirillum* spp., and *Methylobacterium* sp., N_2_-fixing endophytic bacteria [19,20,21].

Inoculation in HS with *Azospirillum* spp. has been carried out on several crops. Mia et al. (2010) found out that *Azospirillum* sp. and *Bacillus* sp. enhanced nitrogen yield in bananas seedlings [22]. A consortium comprising several nitrogen fixers, such as *Azotobacter chroococcum*, *Azospirillum brasilense*, *Pseudomonas fluorescens*, and *Bacillus subtilis*, developed by Aini et al. (2019) [23], doubled the intake of nitrogen. Last but not least, a hybrid culture of unknown nitrifiers and ammonifiers was able to use organic nitrogen in a tomato crop, for hydroponic farmers to convert from conventional inorganic fertilizers to organic ones [24].

Some PGPB have the capacity to produce indole acetic acid (IAA), which enables them to increase root surface area and promote in plant development [14]. It has been often proved that microbial inoculants producing IAA, such as *Azospirillum* sp., have increased roots hair growth and roots biomass [13] in soil condition. In addition, inoculation of crops grown in HS with IAA-producing PGPB has been a way to enhance yields, shortening the acclimatation period and reducing abiotic stress [25]. Moghaddam [26] proved that radish grown in HS could increase root number, dry weight, root length, and wet weight when inoculated with IAA-producing *Azospirillum* sp. Furthermore, El-Kawas [27] registered that *Azospirillum* sp., and *Klebsiella* sp., cell-free supernatant (containing IAA produced by the microbial strains) increased root development in rice plants in HS.

*Bacillus* spp. are highly employed as PGPB as well, not only as biocontrol agent and abiotic stress resistance inducer [28,29,30]. In HS, *Bacillus subtilis* is widely known for its capacity to reduce resistance to excessive salt concentrations in nutrient solutions, and for promoting plant growth [31,32]. *Bacillus amyloliquefaciens* has been demonstrated to improve tomato water use efficiency and quality in terms of a greater amount of vitamin C [33]. *Bacillus licheniformis* has been shown to improve tomato and pepper weight and diameter, as well as higher yields of each crop [34]. Even if *Bacillus* sp. are known to also be IAA producers, no study has been carried out yet to investigate *Bacillus* sp. ability to produce IAA in HS and its influence on tomato plants growth parameters.

In this research, the aim was to investigate the impact of seven IAA-producing bacterial strains from the genera *Azospirillum*, *Bacillus*, and *Methylobacterium* on the early growth stages of tomato (*Solanum lycopersicum* L.) plantlets in HS, developing a novel hydroponic system specifically designed to facilitate the study of plant–microbial interactions.

## 2. Materials and Methods

### 2.1. Microbial Strains

Seven bacterial strains and two mixes were used as listed in Table 1. Polymicrobial inoculants (MIX A and MIX B) were prepared accordingly to past preliminary results (pers. comm.) of a dual-culture assay (Cross Streak Assay using Living Cells) [35]. Stock cultures were stored at −80 °C in 20% glycerol and before being used, they were grown overnight at 27 °C at 120 rpm in liquid Nutrient medium: Lab-Lemco powder 1.0 g/L; yeast extract 2.0 g/L; peptone 5.0 g/L; sodium chloride 5.0 g/L (Oxoid, Thermofisher, Milan, Italy). 

### 2.2. Preparation and Germination of Tomato Seeds

Tomato seeds (No 550) of variety ‘Canestrino di Lucca’, provided by Gargini Sementi di Toscana s.n.c. (Lucca, Italy), were surface sterilized by immersion in 70% ethanol for 1 min, followed by treatment with 10% hypochlorite for 5 min, and subsequently rinsed five times in sterile distilled water.

The described approach intends to provide a support to seedlings at very early stage of growth using metal mesh structures, while simultaneously co-cultivating them alongside bacteria: it is a modification of the work provided by Nathoo et al. [41].

**Figure 1:**
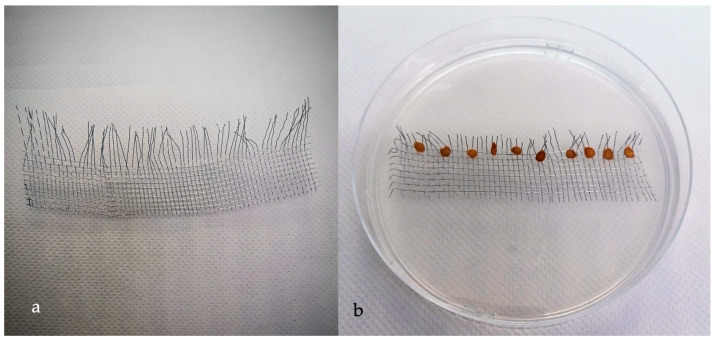
(**a**) Metal mesh combs (MMC) were prepared by cutting strips of 2 cm × 12 cm × 1 mm with common scissors, and some strings from the long side were removed to shape the mesh like a comb; (**b**) one single sterilized MMC, containing 10 sterilized seeds, was placed on the water-agar in Petri dish.

First, metal mesh combs (MMC) were prepared. Strips of 2 cm × 12 cm × 1 mm were cut with common scissors. Then, some strings from the long side were removed to shape the mesh like a comb (Figure 1a). Once prepared, MMC were autoclaved at 121 °C for 20 min. Petri dishes (dia. 120 mm, N = 50) containing sterilized water-agar (10 g/L, Agar Technical—Biolife, Milan, Italy) were prepared. One single sterilized MMC was placed on the solid agar of each Petri dish. Finally, 10 sterilized seeds per Petri dish were placed on the MMC as shown in Figure 1b and closed with lids and Parafilm^®^ tape. This step, timepoint 0 (T0), was considered the beginning of the experiment. Thus, Petri dishes were placed at 4 °C for 24 h in the dark. Afterwards, the seeds were left germinating at 22–24 °C in the dark for 5 days (T5). At T5, the germination rate was recorded as percentage of germinated seeds [42].

### 2.3. Acclimation

**Figure 2:**
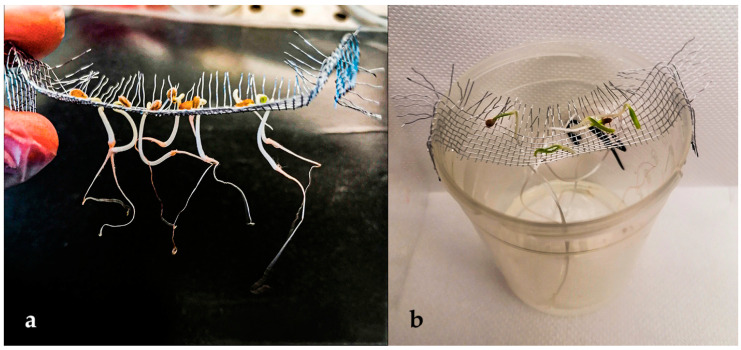
Hydroponic co-cultivation system: the metal mesh combs (MMC) enabled (**a**) collecting all the plantlets at the same time with one single motion, and (**b**) extending into the liquid solution the roots of seedlings.

The seedlings needed to go through an acclimatization period that enabled the seedlings to adapt to the hydroponic environment before inoculation with microorganisms. Acclimation was performed by moving the seedlings into sterile clear plastic jars (100 mL) and enabling the seedlings to grow for 3 days using only Hoagland solutions (Figure 2). Two different Hoagland solutions were prepared, according to Kaur et al., 2016 [43]: HT (Hoagland Total) was set up for the inoculation for *Bacillus* spp. and MIX B, and HN (Hoagland for Nitrogen-fixers) solution was set up for the inoculation with the nitrogen-fixing bacteria (*Azospirillum* spp., *M. symbioticum* and MIX A [44,45,46]). Hoagland Total (HT) contained Ca(NO_3_)_2_·4H_2_O, KNO_3_, KH_2_PO_4_, MgSO_4_·7H_2_O, trace elements, FeEDTA—all nutrients; Hoagland for nitrogen-fixers (HN) excluded nitrogen sources (Ca(NO_3_)_2_·4H_2_O and KNO_3_). The decision to use two types of Hoagland solutions—one with all nutrients (HT) and one identical but without nitrogen (HN)—is based on the different metabolic needs of microorganisms and the response of plants to stress. When plants experience nitrogen deficiency, they produce root exudates to attract beneficial microorganisms [47,48]. A lack of nitrogen triggers nitrogen fixation processes in bacterial strains and encourages plants to form beneficial relationships with the bacteria [47]. According to Souza, Saijai, and Kontopoulou [44,45,46], this approach is commonly used in such experimental setups. Both solutions were prepared at half-strength and supplemented with 1.5 g/L of L-tryptophan, to favor the production of auxins by the bacterial inoculants [49,50]. The jars were filled with 80 mL of the respective Hoagland solution in a sterile flow hood. A total of 30 jars were filled with HN solution, while 25 were filled with HT solution. The MMC enables the roots of the seedlings to extend into the liquid solution. The jars were sealed with lids, and the seedlings were incubated at 22–24 °C for 72 h (T8). Shaking at 50 rpm was used to provide aeration.

### 2.4. Co-Inoculation

Microbial cell suspensions were prepared according to the method described by Bartolini et al. [51]. The bacterial cells were then separated from the supernatant by centrifugation at 4000 rpm for 7 min. The supernatant was carefully removed, and the cells were resuspended in HN or HT solution to obtain a concentration of 1 × 10^8^ CFU per 80 mL. *Azospirillum baldaniorum* Sp245, *Azospirillum brasilense* Sp7, *Azospirillum brasilense* Cd, *Methylobacterium symbioticum* SB0023/3T and MIX-A were inoculated with the HN solution, and *Bacillus amyloliquefaciens* (Fukumoto strain F), *Bacillus licheniformis* (Gibson 46), *Bacillus subtilis* 101BS, MIX-B with the HT solution. The control groups were represented by un-inoculated HN (for C_(a)_) and HT (for C_(b)_). Tomato seedlings in the HS were cultivated for 10 days under natural light at 22–24 °C, shaking at 50 rpm to provide aeration.

**Figure 3:**
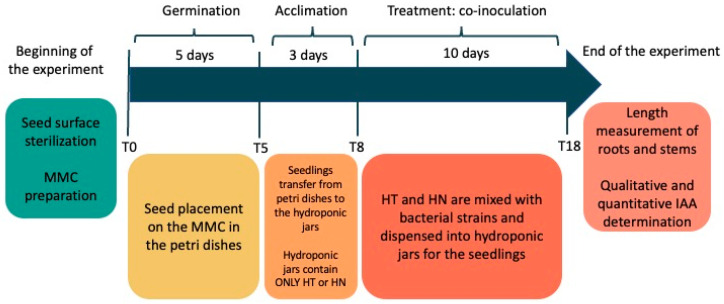
Timeline of the hydroponic co-cultivation system experiment. T0: beginning of the experiment; T0–T5: germination; T5–T8: acclimation (plantlets were moved from Petri dishes to hydroponic jars, containing only the HN or HT solutions). T8–T18: co-inoculation (microbial cell suspensions are added to the hydroponic jars). T18: end of the experiment.

The tomato seedlings were taken out of jars. The length of stems and roots was measured at the beginning of the co-inoculation phase (T8) and at the end of the experiment (T18), using a software for image processing (ImageJ-Version 1.54g-Rasband, W.S., U.S. National Institutes of Health, Bethesda, MD, USA, https://imagej.net/ (accessed on 18 October 2023) 1997–2018). At T18, the survival rate of the seedlings has also been registered. The temporal timeline of the experiment is schematically shown in Figure 3.

### 2.5. Analysis of Indole-3-Acetic Acid (IAA) Production

At the conclusion of the co-cultivation phase, when the plantlets were removed from the jars, both the plantlets and the remaining HT and HN solutions were tested for the presence of auxin (IAA) produced by each bacterial strain and mixtures. In these experiments a further control group containing IAA without the presence of bacteria was omitted considering that, previous preliminary trials (pers. comm.), did not give effective results on the survival rate and root architecture.

A colorimetric assay using the Salkowski reagent method, as described by Gang et al. [50], with modifications. Salkowski’s reagent comprises a solution containing 35% HClO_4_ and 10 mM FeCl_3_, which reacts with IAA (indole-3-acetic acid) to form a tris-(indole-3-acetate) iron (III) complex, resulting in a pink coloration.

To achieve the in situ staining of the plantlets’ organs, the roots and stems of tomato seedlings were stained dipping the plantlets in 500 μL of Salkowski reagent for 30 min, as described by Gang et al. [50].

The quantification of auxins was conducted on the inoculated HN and HT solutions at the conclusion of the experiment, following the method described by Gang et al. [50].

Quantification was performed using the Infinite^®^ M Nano microplate reader (Tecan, Männedorf, Switzerland) with a 96-well plate. Auxin levels expressed as mg/L were determined by spectrophotometric assay at 530 nm. All determinations were performed in triplicate, including control groups and the calibration curve.

### 2.6. Data Analysis

Statistical analyses have been performed using both R Statistical Software (v4.1.2; R Core Team 2021) and the package GraphPad Prism 10 (GraphPad Software, Inc., version 10.0.0 for Mac OS X, Boston, MA, USA, www.graphpad.com). Prior to analyses, data were log, square-root and square-transformed to satisfy normality and homoscedasticity assumptions. The standard errors (±SE) of the means were calculated for each parameter measured, considering *p* ≤ 0.05. Data were compared using analysis of variance (ANOVA) and Tukey’s multiple range test was assessed to compare the differences among means.

## 3. Results

### 3.1. The Germination and Survival Rate of Tomato Seeds

The germination rate of Canestrino tomato seeds, maintained on MMC in a water- agar substrate, was assessed after 5 days (T5). At this time, a germination average (Avg) around 77% was recorded.

At the experimental ending (T18), the survival of the seedlings was recorded, and a marked difference between bacterial co-inoculation treatments and controls was observed. The mean survival rate of the treated seedlings was approximately 95%, whereas the control plants had a significantly lower mean survival rates (66% for C_(b)_ and 50% for C_(a)_). Among the tested bacteria, *A. baldaniorum* Sp245 showed the highest rate, making 98.5% of plantlets survive (Table 2).

### 3.2. Effect of the Bacterial Strains Inoculation on Roots and Stems

**Figure 4:**
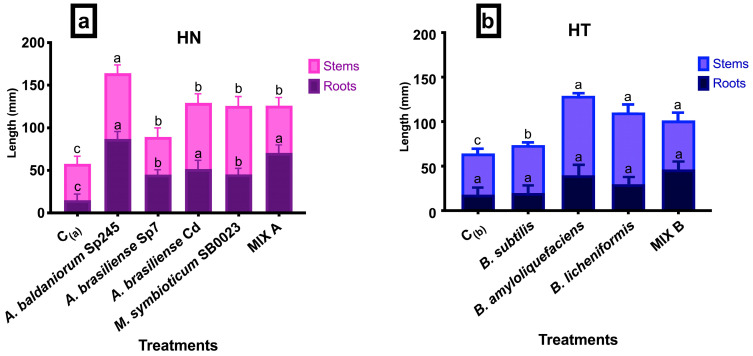
Mean length (±SE) of roots and stems of tomato plantlets treated with: (**a**) *Azospirillum* spp., *M. symbioticum*, MIX A in HN (Hoagland for N_2_ fixers) solution; (**b**) *Bacillus* spp., MIX B, in HT (Hoagland Total) solution. Controls: C_(a)_ and C_(b)_. Different letters among roots and stems indicate significant differences at *p* ≤ 0.05.

At the end of the experimental period (T18), in which tomato plantlets were subjected to the bacterial co-inoculation treatments that lasted 10 days, plantlets were collected from the hydroponic system, and the length of roots and stems were measured. In general, all bacteria (alone and mixed) were significantly effective when compared to the control groups (Figure 4a,b). The average increases in the total length of roots and stems ranged from approximately 106% to 175% for HN, and from approximately 56% to 102% for HT. Specifically, plantlets of the control groups showed average length values of 14.97 mm and 17.99 mm for roots, and 42.60 mm and 53.97 mm for stems in HN and HT, respectively. Analyzing the proportional data between hypogeal and epigeal organs, most bacteria from both Hoagland solutions, induced a major development of the stems. Within HN solution (Figure 4a), *A. baldaniorum* Sp245, *A. brasilense* Cd and *M. symbioticum* SB0023 proved to be the best, inducing roots growing of a mean length of 86.92 mm, 51.67 mm and 45.28 mm and stems of a mean length of 77.08 mm, 77.50 mm and 80.50 mm, respectively. Within HT solutions (Figure 4b) the finest were *B. amyloliquefaciens* and *B. licheniformis*, that induced, respectively, a mean root growth of 39.99 mm and 29.99 mm, and a mean stem length of 89.02 mm and 80.55 mm, respectively. It is interesting to note that both MIX (A and B) induced a more equilibrated root and stem growth, closely to 50% of the total. MIX A induced a mean roots length of 70,55 mm and a mean stem length of 55.43 mm; MIX B induced a mean root length of 46.38 mm and a mean stem length of 55.96 mm.

A fundamental difference was observed between the two sets of treatments: bacterial strains from the HN set significantly affected the root length of the plantlets compared to C_(a)_, while the bacterial strains from the HT set did not significantly affect the root length of the plantlets compared to the control group C_(b)_. On the contrary, the HT treatment set was more effective on the stem length.

### 3.3. Analysis of Indole-3-Acetic Acid (IAA) Production

**Figure 5:**
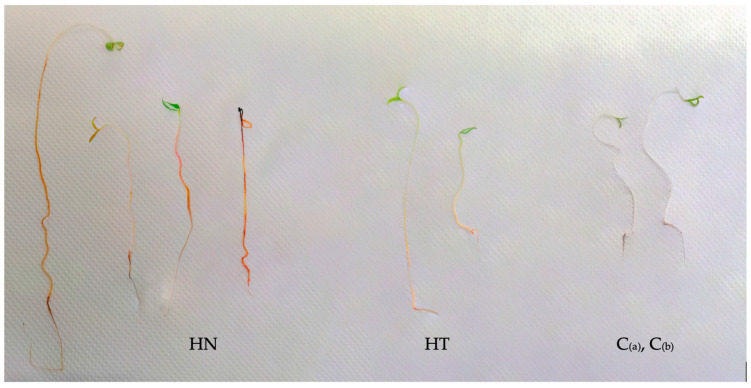
In situ Salkowski reagent staining on tomato plantlet organs treated with microbial strains added to different Hoagland solutions: HN (Hoagland for N_2_ fixers) and HT (Hoagland Total for *bacillus*). The appearance of a pinkish-red hue on roots and stems shows the presence of auxin. In contrast, control plants (C_(a)_ and C_(b)_) lack any pink coloration.

**Figure 6:**
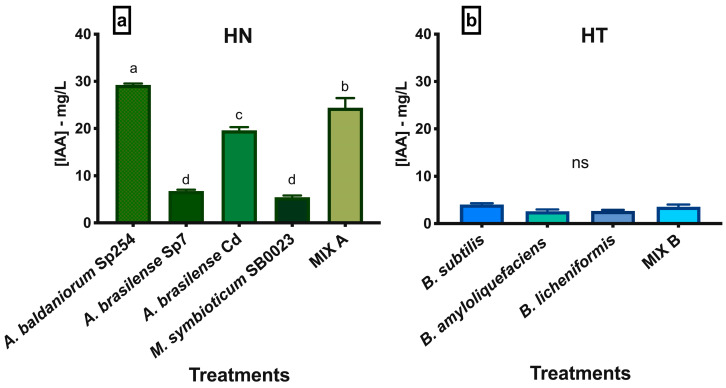
Mean IAA concentration (±SE) synthetized by each strain in the HN solution (**a**) or in the HT solution (**b**) at the end of the experiment. Different letters indicate significant differences at *p* ≤ 0.05; ns (not significant).

Results obtained from an IAA qualitative assessment by the Salkowski’s reagent test showed that each examined strain was able to metabolize L-tryptophan (TRP) into IAA or similar compounds (Figure 5 and Figure 6). The visual evaluation of a pinkish-red hue on roots and stems of tomato plantlet enabled identifying the spatial distribution of a probable IAA synthesis induced by the microbial strains. A difference in staining intensity was observed among the different treatments, in comparison with control plants that were lacking in pink appearance after the Salkowski’s reagent application (Figure 5). In particular, *Bacillus* spp. exhibited less intense staining, while *Azospirillum* spp. showed the strongest intensity.

The quantification of auxins production revealed that the presence of microbial strains in hydroponic solutions, either alone or in combination, was responsible for the synthesis of IAA compounds. Indeed, IAA was not detected in the control groups (C_(a)_ and C_(b)_), consisting of un-inoculated HN and HT), confirming results of the visual observations.

About bacteria added to HN solution (Figure 6a), IAA concentrations ranged between 3.56 mg/mL (*M. symbioticum*) and 29.23 mg/mL (*A. baldaniorum* Sp245). In addition to the latter, other valuable IAA-producing strains were *A. brasilense* Cd (19.61 mg/L) and MIX A (24.42 mg/L).

*Bacillus* spp. strains were less prone to produce IAA (Figure 6b): *B. subtilis* produced 4.03 mg/L, *B. licheniformis* 2.69 mg/L and *B. amyloliquefaciens* 2.63 mg/L. The mix of the *Bacillus* spp. strains (MIX B) produced 3.56 mg/L.

**Figure 7:**
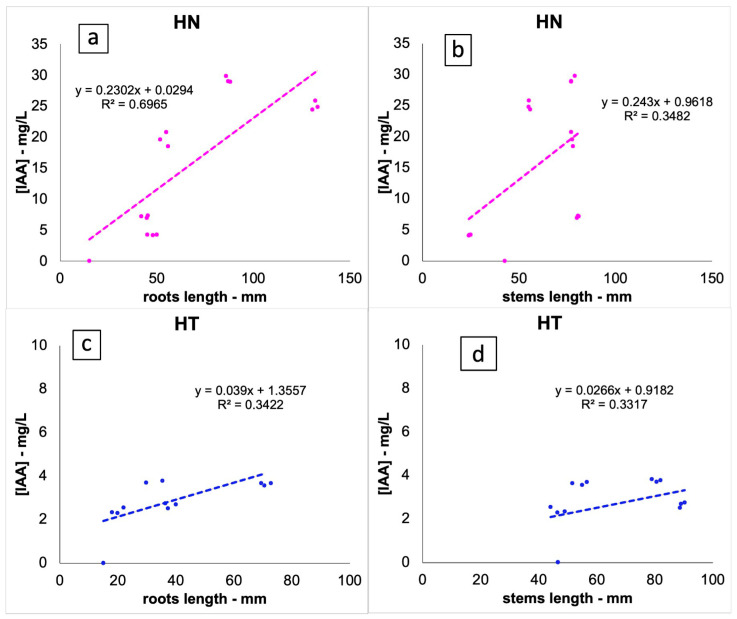
Linear regression analyses correlating IAA production by the bacterial strains from the HN (**a**,**b**) and HT (**c**,**d**) set with root and stem length.

Furthermore, a regression analysis (Figure 7) was carried out to examine the relationship between IAA produced by the bacterial strains and the metric parameters of the plants. Concerning roots, the results revealed a high positive correlation (R^2^ = 0.6965) between IAA production and length of roots from the HN set (a).

## 4. Discussion

The seed germination is a critical stage of the life cycle of a tomato plant. Thus, the seed quality is crucial for a successful cultivation [52,53]. Tomato seeds of Canestrino variety showed good germination performance in terms of time and germination rate in accordance with what was declared on the label by the manufacturer. Although the percentage shown on the label (90%) is referred to as the germination rate of seeds in the soil, it is encouraging that the obtained rate was approximately 77%. A 77% germination is not a high absolute rate, but it is consider encouraging since the seeds were not left germinating in a condition that is considered standard as suggested by the producer (soil or a wet paper towel), but in a possible “hostile” situation (MMC + water-agar). This result suggests that the methodology here proposed, based on a water-agar germination substrate, with the MMC (metal mesh combs) structure, did not negatively impact seed germination.

The MMC, set up for the experimental trials, proved to be effective for easy collection of plantlets after germination, and for the transfer from the germination culture to the hydroponic co-cultivation system. This is an innovative arrangement inspired by the work of Nathoo et al. (2017) [41]. This method enabled treating tomato plantlets in a hydroponic system (HS) adding different bacterial strains to the nutrient solutions. In relation to the different characteristics of bacteria, plants can increase the ability to engage symbiotic relationships with microorganisms [54,55,56]. In particular, the lack of synthetic nitrogen supply in HS can be successfully overcome by the presence of nitrogen-fixing bacteria in the nutrient solution [46]. The significant improvements of the tomato plantlets’ growth metrics when placed in HN with *A. baldaniorum* Sp245 and *A. brasilense* Cd were in agreement with Setiawati [57]. who attributed this finding to the ability of *Azospirillum* spp. to fix atmospheric N_2_, in addition to synthesize phytohormones, such as indole-3-acetic acid (IAA). IAA is a crucial phytohormone-like substance playing a significant role in root-microbe interactions: it enhances the quality of root system architecture and promotes the elongation of the tissues [49]. Coherent results were obtained on rice roots by Moghaddam [26] although it was used cell-free supernatant from an IAA-producing *Azospirillum* sp. Furthermore, Burdman also [58] proved that *A. brasilense* Cd inoculation in HS *Phaseolus vulgaris* enhanced root hair formation. In this context, consistent results were obtained for HN set as confirmed by the correlation analyses showing a significant relationship between the root elongation of the tomato plantlets and the IAA amount found in the nutrient solution.

Due to the ability of bacteria to produce different compounds, HT set for *Bacillus* spp. inoculation showed a poorer growth of the roots’ length, but an improved growth of the stems, in agreement with several authors [59,60]. In fact, *Bacillus* spp. are known to also be producers of other phytohormones, such as gibberellic acid [60], which, among other functions, is responsible for inducing stem growth in plants [61].

A positive influence on root growth was also found for *M. symbioticum*, isolated in 2020 [21] and characterized only as an N_2_ fixer, whose foliar application has been successfully proved on strawberry, maize and lettuce [21,62,63]. Our investigations about the employment of *M. symbioticum*, both alone and in combination with IAA-producing PGPB, appeared promising and innovative. Among other things, its presence in HS determined a good survival rate of the tomato plantlets, similar to the other bacteria. Thus, *M. symbioticum* could have a potential role as bio-inoculant suitable to be employed at early-stage growth of seedlings, particularly as part of a consortium of IAA-producing PGPB.

Recent researches have highlighted the benefits of polymicrobial inoculations in promoting synergistic or complementarity interactions among different microbial strains, leading to a more stable, resilient and functional growth-promoting effect on the crops [64]. Cerozi et al. (2016) using a commercial mixture of *Bacillus* spp., have found positive influences on ammonia, nitrite, and nitrate levels in a nutrient film technique (NFT) system with Red Cherokee lettuce [65]. Furthermore, on a study involving Tiberius romaine lettuce, a consortium of nitrogen-fixing bacteria, including *Azotobacter chroococcum*, *A. brasilense*, *Pseudomonas fluorescens*, and *B. subtilis* has been utilized which improved nitrogen fixation [23]. Also, two mutated strains of *A. brasilense* (Sp7 and Sp245) exhibited higher nitrogenase activity when inoculated together on wheat plants in a HS [66] and a mix of *A. brasilense* strains REC2 and REC3 proved to have fungicide activity on HS cultivated strawberry against *Colletotrichum acutatum* [67]. Moreover, co-culturing tomato seedlings with both wild-type and transformed *A. brasilense* Cd and *Chlorella vulgaris* positively affected the stem length in high salinity HS [68].

The significant growth enhancement and a better root-to-stem balance of tomato plantlets recorded after both polymicrobial addition (MIX A and MIX B) to our innovative HS, suggests the occurrence of a synergistic effect among the microbial strains. This confirms the potential of polymicrobial inoculants to optimize plant growth and health in HS.

This kind of system (MMC + PGPB) could be employed for many purposes: it could be adapted for different crop plantlets in the early stage of growth in order to study plant–microbial interaction. Additionally, in this system, microbial inoculants can be crafted and scaled up for industrial HS, to meet the specific needs of different crops.

The primary challenge microbial inoculants face in HS is similar to the one they face in soil applications: inoculants might overcome to more competitive strains or microbial populations in the nutrient solution. This issue can be mitigated by developing synthetic polymicrobial inoculants that include both PGPB strains and biocontrol strains. Such combinations can promote plant growth while controlling the proliferation of undesirable microorganisms.

With proper research, the possibilities are vast. Environmentally sustainable and organic HS can become an accessible reality to horticultural sectors.

## 5. Conclusions

In the present work, seven auxin-producing endophytic PGPB strains (*Azospirillum* spp., *Methylobacterium symbioticum*, *Bacillus* spp.) and two different combinations of these strains were applied to tomato during germination and development in hydroponic conditions. The hydroponic co-cultivation system described in this paper provides a solid and easy-to-build system that enables inoculating plantlets, at an early stage of growth (5 days old), with microbial suspensions. Tomato plants were used as model plant, but the system is very flexible and can be adjusted to work with many different kinds of plants. PGPB strains that are known to produce IAA were used, but after an extensive review of literature, it can be stated that no prior study has measured the amount of IAA produced by these particular strains, nor has a study used them in a HS. Results obtained with the MIXes are fundamental: polymicrobial inoculants have demonstrated their superior ability to induce plant growth effectively. Identifying successful polymicrobial inoculants can be beneficial for various applications, including soil-based systems. These inoculants use synergistic interactions and functional complementarity, disrupting competition in the soil and providing multiple benefits to plants. The promising results of this study suggest that further exploration and characterization of polymicrobial inoculants can lead to significant advancements in agricultural practices, although further studies and case-by-case assessments are needed.

This novel model could be profitable employed and opportunely modified to fit the experimental purposes and with the different plant morphologies. This system could be employed to explore the broader applications and plant growth promotion properties of bacterial strains.

## Figures and Tables

**Table 1:** Microbial strains employed in this research.

Species	Strain	Reference/Source	Isolated from
*Azospirillum baldaniorum*	Sp245	Baldani et al., 1986 [36]; Dobbelaere et al., 1999 [37]; dos Santos Ferreira 2020 [38]	*Triticum aestivum*—Brazil
*Azospirillum brasilense*	Sp7	DSMZ *	*Digitaria decumbens* roots—Brazil
*Azospirillum brasilense*	Cd	DSMZ	*Cynodon dactylon* roots—USA
*Bacillus amyloliquefaciens*	Fukumoto strain F	DSMZ	soil—unknown county
*Bacillus licheniformis*	Gibson 46	DSMZ	country of unknown origin
*Bacillus subtilis*	101BS	Filippi et al., 1987 [39]; Citernesi et al., 1994 [40]	carnation rhizosphere
*Methylobacterium symbioticum*	SB0023/3T	Pascual et al., 2020 [21] Symborg Inc. (Murcia, Spain) ** (EP Application No. EP3747267A1)	spores of *Glomus iranicum*—Spain
MIX A	*Azospirillum baldaniorum* Sp245; *Azospirillum brasilense* Sp7; *Azospirillum brasilense* Cd, *Methylobacterium symbioticum* SB0023/3T		
MIX B	*Bacillus amyloliquefaciens* Fukumoto strain F, *Bacillus licheniformis* Gibson 46, *Bacillus subtilis* 101BS		

* DSMZ—German Collection of Microorganisms and Cell Cultures (GmbH, Braunschweig, Germany). ** Symborg Inc., Avenida Jesús Martínez Cortado, 51 30100-Espinardo, Murcia (Spain).

**Table 2:** Mean survival rate (±SE) of Canestrino tomato seedlings at the end of the co-inoculation treatments (T18) with different bacteria. Different letters indicate significant differences at *p* ≤ 0.05.

Treatment	Survival (%)
*Azospirillum baldaniorum* Sp245	98.5 ± 0.5 a
*Azospirillum brasilense* Sp7	92.4 ± 1.2 a
*Azospirillum brasilense* Cd	95.2 ± 2.4 a
*Methylobacterium symbioticum* SB0023/3T	95.1 ± 1.5 a
MIX A	96.8 ± 1.7 a
*Avg*	95.6 ± 1.8
Control	50 ± 1.1 b
*Bacillus amyloliquefaciens*	91.6 ± 0.4 a
*Bacillus licheniformis*	88.9 ± 1.0 a
*Bacillus subtilis* 101BS	89.2 ± 1.2 a
MIX B	90.1 ± 1.3 a
*Avg*	89.9 ± 1.1
Control	66.1 ± 2.2 b

## Data Availability

Datasets analyzed during the study are available from the corresponding author.
